# Diffuse Myocardial Fibrosis detected by Multi-slice T_1_ Mapping using Slice Interleaved T_1_ (STONE) Sequence in Patients with Hypertrophic Cardiomyopathy

**DOI:** 10.1186/1532-429X-18-S1-P238

**Published:** 2016-01-27

**Authors:** Shingo Kato, Steven Bellm, Sébastien Roujol, Jihye Jang, Tamer Basha, Sophie Berg, Kraig V Kissinger, Beth Goddu, Martin Maron, Warren J Manning, Reza Nezafat

**Affiliations:** 1 grid.239395.70000000090118547Beth Israel Deaconess Medical Center, Boston, MA USA; 2 grid.268441.d0000000110336139Yokohama City University, Yokohama, Japan; 3 grid.67033.310000000089344045Tufts Medical Center, Boston, MA USA

## Background

The presence of myocardial fibrosis is associated with worse clinical outcome in hypertrophic cardiomyopathy (HCM) patients. Due to the substantial variations in left ventricular (LV) wall thickness and fibrosis in HCM, volumetric coverage of entire LV myocardium is essential for the accurate assessment of myocardial fibrosis. Slice-interleaved T_1_ (STONE) mapping sequence allows for the assessment of native T_1_ time with complete coverage of LV myocardium. The aim of this study was to investigate whether STONE sequence is useful for the assessment of regional variability of LV native T_1_ time in HCM patients.

## Methods

Twenty-four septal HCM patients (56 ± 16 years) and 10 healthy adult control subjects (57 ± 15 years) were studied. Native T_1_ mapping was performed using STONE sequence which enables acquisition of 5 slices in the short-axis plane within a 90 sec free-breathing scan. The sequence was acquired in a free-breathing ECG-triggered slice-selective bSSFP with the following parameters: 5 slices, in-plane resolution = 2.1x2.1 mm^2^, slice thickness=8 mm, slice gap=4 mm, field of view=360x352 mm^2^, TR/TE/α=2.8 msec/1.4 msec/70 ;, SENSE-factor=2, linear ordering, 10 linear ramp-up pulses and acquisition window=240 msec. We measured LV native T_1_ time and maximum LV wall thickness in each 16 segments from 3 slices (basal-, mid- and apical-slice). Late gadolinium enhanced (LGE) MRI was acquired to assess presence or absence of myocardial enhancement.

## Results

In HCM patients, LV native T_1_ time was significantly elevated compared to healthy controls, regardless of presence or absence of LGE (mean native T_1_ time; LGE (+) segments (n = 27), 1139 ± 55 msec; LGE (-) segments (n = 351), 1118 ± 55 msec; healthy control (n = 160),1065 ± 35 msec; p < 0.001 by one-way ANOVA, 6 segments were excluded from analysis due to artifacts). Among 351 segments without LGE, native LV T_1_ time was diffusely elevated over the 16 segments (Figure). Significant positive correlation was found between LV wall thickness and native LV T_1_ time (y=1013+8.7x, p < 0.001).

## Conclusions

In HCM, substantial number of segments without LGE showed elevated native T_1_ time, and native T_1_ time was correlated with LV wall thickness. Slice-interleaved T_1_ mapping by using STONE sequence could be advantageous to overcome limited cardiac coverage of conventional single-slice T_1_ mapping technique and to accurately detect the diffuse myocardial fibrosis in HCM patients.

**Figure 1:**
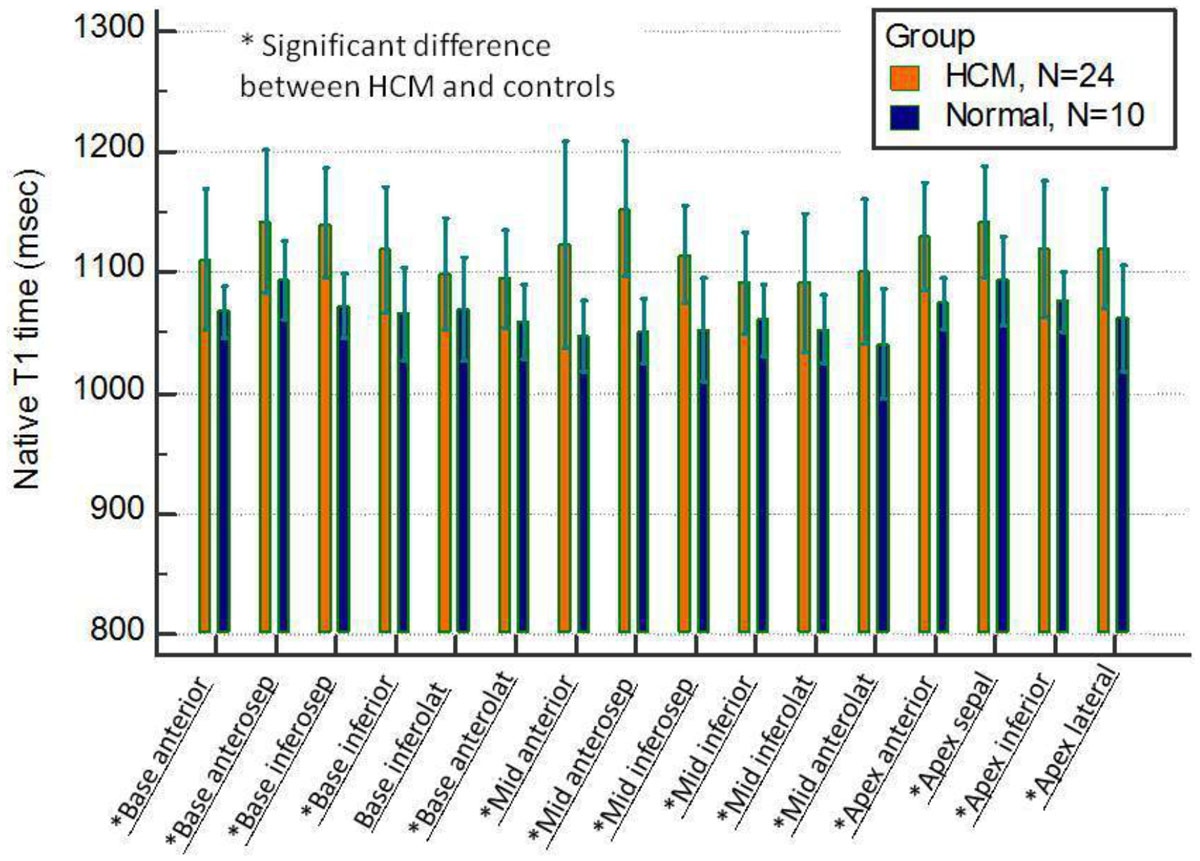
**Comparison of native T**_**1**_ **time between HCM and controls (segments without LGE)**.

